# Influence of cathode geometry on electron dynamics in an ultrafast electron microscope

**DOI:** 10.1063/1.4994004

**Published:** 2017-07-17

**Authors:** Shaozheng Ji, Luca Piazza, Gaolong Cao, Sang Tae Park, Bryan W. Reed, Daniel J. Masiel, Jonas Weissenrieder

**Affiliations:** 1 KTH Royal Institute of Technology, Material Physics, Electrum 229, SE-16440 Kista, Sweden; 2 Integrated Dynamic Electron Solutions, Inc. (IDES), Pleasanton, California 94588, USA

## Abstract

Efforts to understand matter at ever-increasing spatial and temporal resolutions have led to the development of instruments such as the ultrafast transmission electron microscope (UEM) that can capture transient processes with combined nanometer and picosecond resolutions. However, analysis by UEM is often associated with extended acquisition times, mainly due to the limitations of the electron gun. Improvements are hampered by tradeoffs in realizing combinations of the conflicting objectives for source size, emittance, and energy and temporal dispersion. Fundamentally, the performance of the gun is a function of the cathode material, the gun and cathode geometry, and the local fields. Especially shank emission from a truncated tip cathode results in severe broadening effects and therefore such electrons must be filtered by applying a Wehnelt bias. Here we study the influence of the cathode geometry and the Wehnelt bias on the performance of a photoelectron gun in a thermionic configuration. We combine experimental analysis with finite element simulations tracing the paths of individual photoelectrons in the relevant 3D geometry. Specifically, we compare the performance of guard ring cathodes with no shank emission to conventional truncated tip geometries. We find that a guard ring cathode allows operation at minimum Wehnelt bias and improve the temporal resolution under realistic operation conditions in an UEM. At low bias, the Wehnelt exhibits stronger focus for guard ring than truncated tip cathodes. The increase in temporal spread with bias is mainly a result from a decrease in the accelerating field near the cathode surface. Furthermore, simulations reveal that the temporal dispersion is also influenced by the intrinsic angular distribution in the photoemission process and the initial energy spread. However, a smaller emission spot on the cathode is not a dominant driver for enhancing time resolution. Space charge induced temporal broadening shows a close to linear relation with the number of electrons up to at least 10 000 electrons per pulse. The Wehnelt bias will affect the energy distribution by changing the Rayleigh length, and thus the interaction time, at the crossover.

## INTRODUCTION

Ultrafast transmission electron microscopy (UEM) is a powerful tool for studying transient processes in materials at combined high temporal and spatial resolutions.^1–7^ In contrast to conventional transmission electron microscopy (TEM) operating with continuous electron beams, UEM uses ultrafast laser pulses to generate, through photoemission, ultrashort electron bunches at the cathode in an electron gun of a modified TEM. The fundamental performance of an UEM, such as its temporal, spatial, and energy resolutions, is mainly determined by the initial state of the photo-emitted electron bunch and its dynamic behavior during propagation in the gun and microscope column, through the sample, and to the detector. Through the selection of appropriate cathode and laser parameters, the initial state of the electron bunch, including the number of electrons, beam size, duration, and energy spread, can be fine-tuned according to the requirement of the desired experiment. An UEM based on a modified thermionic emission TEM geometry is a versatile instrument that may be operated for imaging, diffraction, and electron energy loss spectroscopy (EELS) studies.^8,9^ It can be used to address a wide variety of scientific problems.^10^ Most thermionic emission TEMs are designed with Wehnelt electrodes that are placed at an adjustable distance from the cathode (the Wehnelt gap). The Wehnelt acts as an electrostatic lens that simultaneously focuses the beam and determines the beam divergence, controls the electric field over the emission area, and suppresses the transmission of electrons emitted from the shank of the thermionic cathode into the column (limits the effective emission area). Unfortunately, these multiple roles of the Wehnelt are in conflict with the UEM performance. The very act of limiting the effective emission area by the Wehnelt also reduces the electric field at the cathode center, which is detrimental to the temporal resolution. However, if the effective emission area is not limited by the Wehnelt, in a tip geometry one risks accepting electrons emitted from the shank of the cathode. The shank-emitted electrons are subject to large aberrations, including substantial path-length differences that greatly worsen temporal resolution. It was recently demonstrated experimentally that shank-emitted photoelectrons and photoelectrons emitted from the flat truncated surface of a conventional cathode geometry arrive at the sample with a relative time delay when operating an UEM with low bias on the Wehnelt electrode.^11^ Thus, in order to perform imaging and diffraction experiments at high temporal resolution, the UEM must be operated with a sufficiently high Wehnelt bias voltage to reject all shank-emitted electrons from entering the limiting aperture of the column. Unfortunately, several experimental studies have found that the temporal resolution deteriorates with increasing Wehnelt bias voltage.^8,11^ It would therefore be of benefit to eliminate all photoemission from the shank of the cathode. This would facilitate UEM analysis already at a combined low Wehnelt bias and small Wehnelt gap and thus result in improved temporal resolution. A potential solution is the use of disc cathodes. In particular, tantalum disc cathodes with a diameter exceeding that of the photo-exciting laser beam have shown promising results.^11,12^ However, during conventional thermionic operation, a large cathode is detrimental to the performance of the microscope. A large emitting area results in a large emittance and a large emittance (when using 100% of the beam) means lower coherence (length) for the same illumination area at the sample. An alternative solution is the use of graphite-embedded cathodes as demonstrated by the Flannigan group.^13,14^

In this paper, we investigate cathodes with a guard ring geometry for completely eliminating all shank-emissions. A guard ring cathode is composed of a central LaB_6_ cylinder surrounded (guarded) by a graphite mantle. Since the work function of graphite exceeds the photon energy of the exciting laser, the emission area is dictated by the size of the LaB_6_ cylinder, not by the suppressing effect of the Wehnelt and not by the laser spot size as for a tantalum disc cathode. Thus, under realistic operating conditions for imaging and diffraction, a guard ring cathode will allow the electric field over the emission area to be both larger and more uniform than in the case of a cathode in the truncated tip geometry. We will demonstrate the performance of the guard ring cathode in UEM and compare to a LaB_6_ cathode of conventional truncated tip geometry to illustrate the impact of cathode geometry on electron dynamics.

Despite the importance of the geometries of the Wehnelt electrode and the photocathode as well as the accelerator region to the overall performance of UEM, surprisingly little has previously been established on what effects dominate the beam quality. In the constant strive for improved temporal resolutions and electron count rates, a thorough understanding of all processes involved during the initial flight path of the electrons must be established. Finite element simulation has been widely used to study electron pulse dynamics in related systems.^15–17^ Recently, Kieft *et al.*^18^ studied the effects of Wehnelt aperture gap and diameter as well as excitation laser-pulse duration on various properties of the photoelectron packet (e.g., collection efficiency, duration, and energy spread) by means of numerical simulations. Here, detailed finite element simulations are employed to describe the influence of the angle distribution of the photoemission process, cathode geometry, local electric fields, as well as Wehnelt bias voltage and gap on the electron beam characteristics. Through a combination of experiments and simulations, we characterize the performance of a guard ring cathode in a thermionic-based photoelectron gun. In comparison to a truncated-tip cathode, the use of a guard ring cathode can allow the Wehnelt to be optimized more freely since it is relieved of much of its duty in limiting the effective emission area and controlling the beam divergence.

## EXPERIMENTAL

**FIG. 1.**
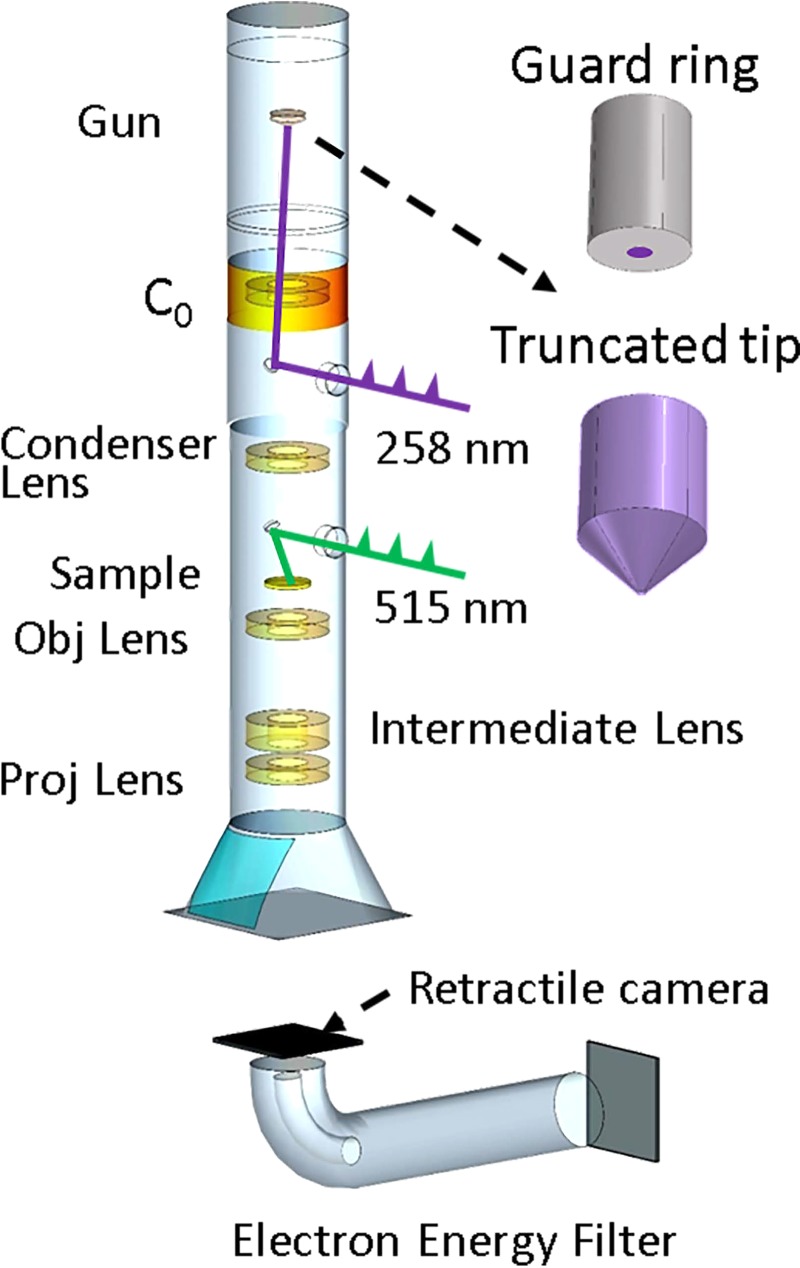
Schematic illustration of the principal elements of the ultrafast electron microscope. Conventional truncated tip and guard ring cathode geometries are shown as insets. The green and purple pulse trains indicate laser pulses for pump and probe (directed to the sample and photocathode, respectively).

The UEM at Material Physics (KTH) is based on a modified JEOL JEM 2100 TEM with a conventional thermionic gun as depicted in Fig. 1. The modification of the UEM was performed by Integrated Dynamic Electron Solutions, Inc. (IDES). Briefly, two vacuum-compatible optical ports were added to the column, allowing optical access to the sample and to the photocathode. Polished aluminum mirrors were installed above the condenser lens system and above the objective lens pole piece, to allow laser illumination of both the photocathode and the sample. Both mirrors are located along the symmetry axis of the TEM column to reduce parallax complications and are fitted with through holes to allow unrestricted electron beam transmission. An additional electron lens (indicated as C_0_ in Fig. 1) is positioned immediately below the anode section. The C_0_ lens may be tuned to collect photoelectrons of a desired divergence angle exceeding the conventional acceptance angle of the JEOL JEM 2100 microscope, increasing the throughput of the photoelectrons into the column. The lens also serves to focus the photoelectrons through the hollow aluminum mirror that directs the laser pulses to the cathode and into the condenser lens.^8,19^ The UEM is equipped with a post column imaging energy filter (Gatan Quantum SE) capable of analyzing the electron energy distribution in both imaging and spectroscopic modes. Laser and related optics are placed on platforms directly coupled to the vibration-isolation system of the TEM column. A magnetic field cancellation system was installed around the TEM to decrease the impact of environmental magnetic fields.

A fiber-amplified laser with a 1030 nm fundamental wavelength and <300 fs pulse duration (Tangerine, Amplitude Systèmes) facilitates the excitation of both the cathode and the sample. The laser has a tunable repetition rate (single shot—2 MHz) and can deliver up to 35 W average power at 100 *μ*J pulse energy. Pulses from a second harmonic generation process (515 nm) are guided through a 400 mm motorized delay stage and directed towards the sample to initiate a transient process. Fourth harmonic generation laser pulses (UV, 258 nm) are directed onto the cathode for the photoemission process. In the experiment, two types of cathodes with different geometries were used to generate the photoelectrons. The first is a conventional truncated tip LaB_6_ cathode with a circular flat area 60 *μ*m in diameter onto which the laser pulse is focused. The second cathode is a guard ring LaB_6_ cathode. This cathode comprises a 50 *μ*m diameter LaB_6_ column surrounded by a 700 *μ*m diameter graphite mantle. The physical shapes of the cathodes are shown as insets in Fig. 1. The high work function of graphite guarantees that essentially no electrons are emitted from the graphite, both under normal thermal emission condition and more importantly during ultrafast photoemission operation. In order to have a similar experimental cut-off voltage (at ∼700 V) for both cathodes, the Wehnelt-cathode gap was set to 700 *μ*m for the guard ring cathode and 600 *μ*m for the truncated tip cathode. The cut-off voltage is the Wehnelt voltage that will completely suppress the electron emission. The cut-off voltage, as well as Wehnelt focusing, is a function of Wehnelt gap, bias voltage, and cathode geometry. It can vary for different cathode types. We also placed the guard ring cathode at 500 *μ*m since the Wehnelt conditions are more relaxed in this geometry (supplementary material, Fig. S4). The bias voltage on the Wehnelt is tuned from 80 V to 700 V.

The dynamics of the electron bunches in the UEM were simulated using the finite element method with a charged particle tracing model on the COMSOL Multiphysics^®^ software platform.^20^ A 3-D model including the Wehnelt, accelerator region, and field free range was implemented in accordance with the real configuration of the present modified TEM. The simulation included both geometries of conventional truncated tip and guard ring cathodes. For a valid comparison between the two cathodes, the flat surfaces where photoemission takes place are both set to 60 *μ*m in diameter in the simulation. Mesh refinement was performed to optimize computational time and accuracy of the results. The propagation of the electrons was simulated by integrating the relativistically corrected Newtonian equations of motion in the calculated electric field, using a generalized alpha time-stepping method. For simulation of space charge effects, the coulombic electron-electron interaction was added during the electron propagation. The FWHM diameter of the UV laser spot illuminating the cathode was assumed to be 100 *μ*m with a Gaussian distribution. Electron packets containing between 1000 and 10 000 electrons with 300 fs Gaussian initial temporal width (based on the laser pulse width) were launched from the LaB_6_ surface of the cathodes to simulate multi-electron bunch dynamics. The initial energy spread was assumed to be of a Gaussian distribution with a mean of 1.2 eV and standard deviation of 0.8 eV and was cut off at E = 0. The photoemission process was assumed to follow Lambertian angular distributions.^21,22^ In the truncated tip geometry, 25% of the photoemission occurred at the truncated flat surface with the remainder being emitted from the shank. The bias voltage on the Wehnelt was tuned from 50 V to 650 V to study the influence of the Wehnelt electrode on the dynamics of the electron bunch.

## RESULTS AND DISCUSSION

This section is divided into subsections with descriptive headings. We begin by reporting the Experimental Results including the temporal duration of electron bunches, the electron energy dispersion, and the effective source distribution. We then continue by describing our Finite Element Simulations including the spatial distribution of electrons during propagation, the temporal spread of electron bunches, and the simulated normalized effective transverse emittance.

### Experimental results

#### Temporal duration of electron bunches

**FIG. 2.**
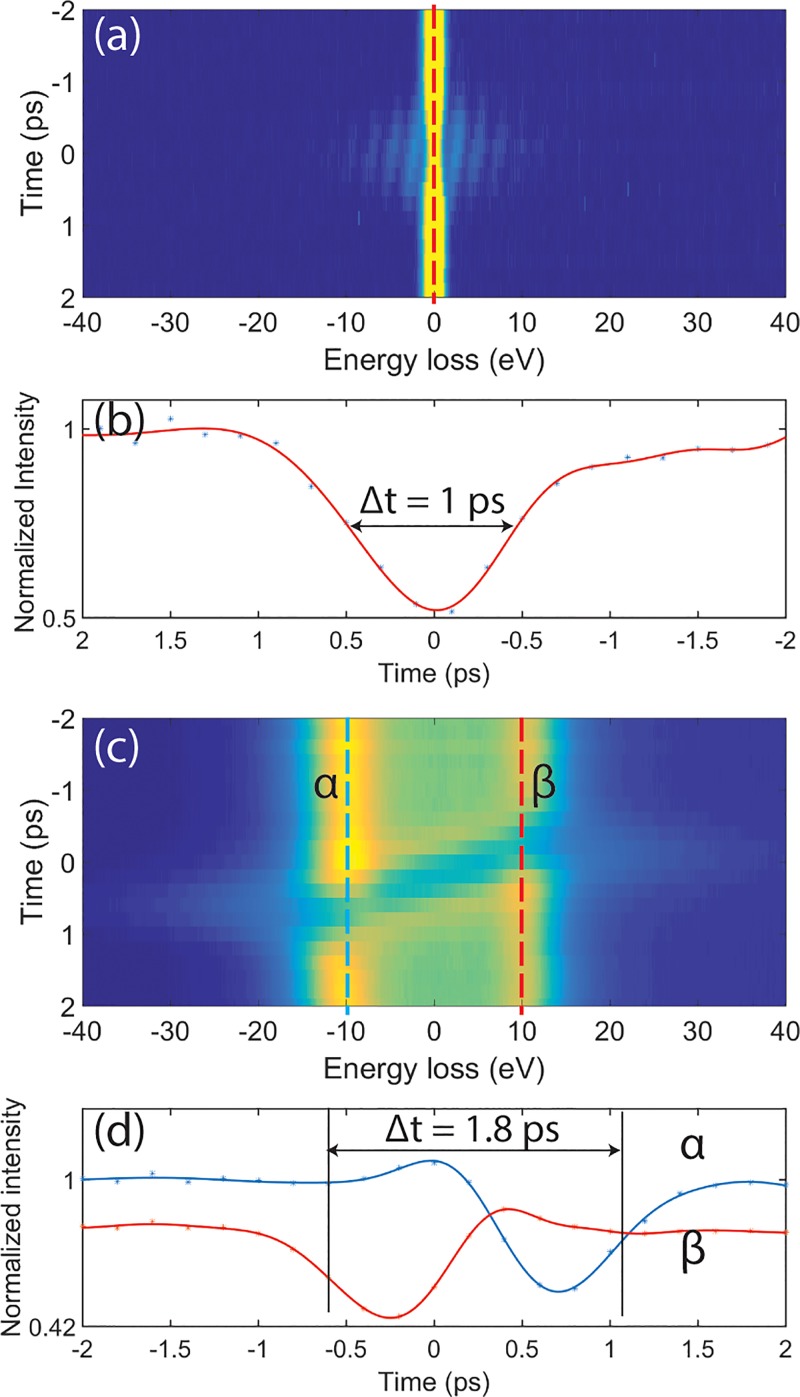
Time series PINEM spectra of silver nanowires using 2.4 eV (515 nm) pump photon energy. **(**a**)** Probed by a few electrons per pulse pulses and **(**c**)** multi-electron pulses (approximately 1000 electrons per pulse at the detector). **(**b**)** The temporal change in intensity of the zero loss peak as indicated by the red dashed line in figure (a). The blue line α and the red line β in **(**d**)** are the temporal intensity changes at ±10 eV electron energy loss corresponding to the indicated blue α and red β lines in image (c).

The temporal duration of the photoemitted electron pulses was characterized by photon-induced near field electron microscopy (PINEM) using a silver nanowires sample.^8^ The PINEM effect is related to the sample geometry. As reported in Ref. 23, surface plasmon polaritons (SPP) may be trapped and propagate on flat, buried, metal/dielectric interfaces. In this geometry, the PINEM signal may become elongated because of the relative long lifetime of the SPP. However, for the case of Ag nanowire, localized surface plasmon can be optically excited directly and can also dissipate quickly as a far field component wave. In this case, the PINEM effect is only present when the exciting laser pulse (pump) and probing electron pulse are spatially and temporally overlapped at the specimen.^24^ In the process, the probe electrons will interact with the evanescent plasmonic near-field generated by the pump laser pulse at the silver nanowires. The interaction may lead to absorption or emission of multiple energy quanta of the pump photon energy.^25^ The resulting gain (and loss) in energy of the electron beam can be accurately measured by the post-column energy analyzer. The intensity of the zero loss peak in the electron energy spectrum is directly related to the cross-correlation of the photon pulse and the electron bunch (in the weak interaction regime).^26^ By changing the relative time delay, the laser pulse will slice the electron bunch from back to front at the sample position. In the low current regime with probe pulses averaging approximately 1 electron at the sample, the energy width of the zero loss peak of the electron beam is smaller than the excitation photon energy and discrete absorption and loss bands, equidistantly positioned by the photon energy, can be observed in the resulting electron energy spectra [Fig. 2(a)]. The change in zero loss peak intensity with time delay is shown in Fig. 2(b). The curve corresponds to the intensity change at the cut indicated by the red dashed line in Fig. 2(a). The full width at half minimum of this profile (about 1 ps) approximately corresponds to the cross-correlation of the laser pulse (300 fs) and the electron bunch at the sample. In the case of multi-electron probe pulses, electron-electron interaction within the bunch, or space charge, will become a significant factor. Space charge will increase the energy dispersion in the electron bunch, and when the width of the zero loss peak in the EELS spectrum exceeds the photon energy of the pump laser pulse, the discrete sidebands are blurred to continuous tails to the distribution. The effect can be observed on both the gain and loss sides of the time scan electron energy loss spectra in Fig. 2(c). Further, since the PINEM effect will transfer the intensity in the electron energy distribution from low to high gain/loss, a sharp decrease in the intensity of the zero loss peak can be observed when the laser pump pulse is at the sample. The positive slope of the depressed region of the spectrum is in line with the interpretation that faster electrons are located at front of the electron bunch and slower electrons at back.^27^ As the laser pump pulse scans through the electron packet from back to front, only the electrons that temporally overlap with laser pulse will emit or absorb energy quanta at the sample position (in line with the PINEM effect). In Fig. 2(c), where 1000 electrons per pulse were detected at the camera, the space charge effect is so severe that the resulting electron energy profile is bimodal: two broad peaks shifted +10 eV and −10 eV on the energy loss scale are evident in the EELS spectra. The peak at −10 eV, corresponding to electrons that have gained energy (faster), is indicated by a blue dashed line α in Fig. 2(c). The +10 eV peak corresponds to the slower electrons and is indicated by a red dashed line β. The intensity of these two peaks as a function of temporal delay is shown in Fig. 2(d). The temporal shift between the time when the first (fast) electrons have reached a position of half the maximum attenuation to the time when the last (slow) electrons are at a position of halfway recovered intensity is here defined as the electron bunch duration [as indicated by the arrow in Fig. 2(d)]. In the present case, the electron bunch length is 1.8 ps.

**FIG. 3.**
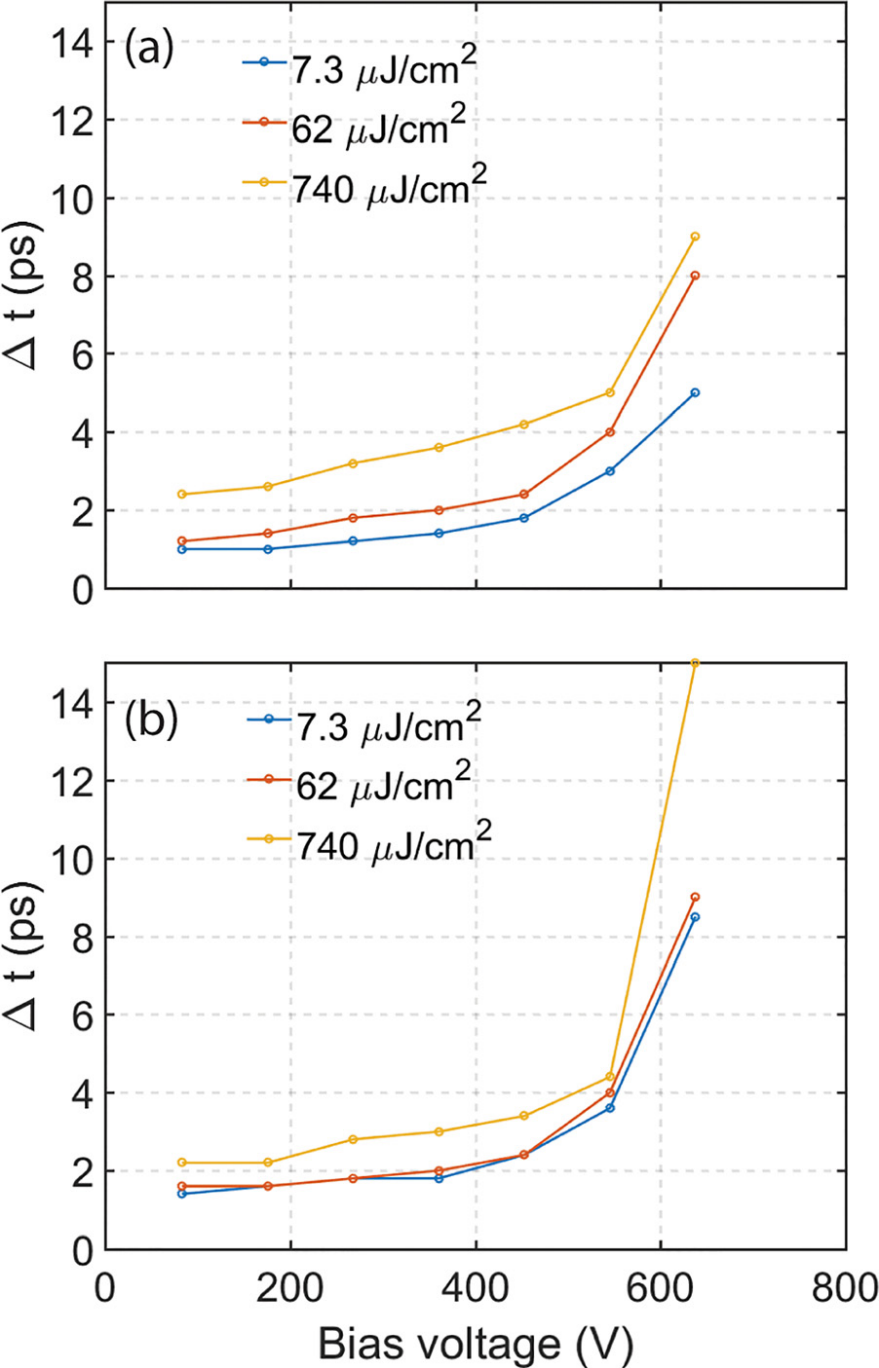
Temporal cross-correlation of the electron bunch and the pump laser as a function of Wehnelt bias and increasing UV pulse energies (blue 7.3 *μ*J/cm^2^, red 62 *μ*J/cm^2^, yellow 740 *μ*J/cm^2^). (a) Guard ring cathode at 700 *μ*m gap and (b) truncated tip cathode at 600 *μ*m gap.

The temporal duration of electron bunches emitted from the guard ring and the truncated tip cathodes was analyzed as a function of Wehnelt bias voltage and exciting laser (UV) pulse energy. The results are shown in Figs. 3(a) and 3(b). Only electrons from the central beam were considered in the analysis of the temporal resolution. The entrance aperture to the EELS spectrometer was used to spatially filter-out the shank-emitted electrons from truncated tip cathodes. It has previously been demonstrated that shank-emitted electrons arrive at the sample with more than 10 ps delay compared to on-axis electrons at low Wehnelt bias voltage.^11^ When operating the microscope in near single electron mode, only a handful of (<10) electrons are detected at the CCD camera for each electron bunch. This mode of operation is achieved for the guard ring cathode at a 7.3 *μ*J/cm^2^ UV laser fluence. The corresponding temporal duration of the cross-correlation [Fig. 3(a)] is approximately 1 ps at 80 V Wehnelt bias voltage for a 700 *μ*m Wehnelt gap. The number of electrons per pulse influences the temporal duration, as shown in Fig. 3(a). The temporal spread of an approximately 1000 electron bunch (as recorded by the retractable camera) generated by a 740 *μ*J/cm^2^ laser pulse exceeds 2 ps at 80 V Wehnelt bias, or roughly twice that of a near single electron bunch. From the same figure, it is apparent that the temporal spread increases dramatically with increasing Wehnelt bias voltage. This holds true for both types of cathodes investigated in the study. The rate of increase of the temporal spread is not linear with the Wehnelt bias. That is, at low Wehnelt bias, a small bias increase will not affect the temporal spread as much as a similar increase at high Wehnelt bias.

#### Electron energy dispersion

The energy resolution in conventional electron microscopy using a thermal emission gun can readily reach values below 1 eV. The resolution is influenced by several factors: the energy width of the electron source, the energy broadening (Boersch effect) arising from electron-electron interaction in the gun region, the electron-optical design of the electron spectrometer, and the stability of the high-voltage and spectrometer power supplies.^28^ In thermal emission mode, after the gun crossover each emitted electron is often assumed to travel down the column at a distance sufficiently far from any other electron such that electron-electron interaction can be ignored at typical beam currents. Thus, with an ideal spectrometer, the Boersch effect near the cathode and thermal broadening will dominate the energy resolution. In conventional thermal emission, Fermi-Dirac statistics yield approximately 0.4 eV spread at 2000 K, a temperature relevant for thermionic emission from a low work function material such as LaB_6_. In practice, 0.6 eV or larger spread is more typical, owing to additional contributions from the Boersch effect and the point spread function of the spectrometer. However, in photoemission mode, the electron generation process is of a completely different nature. The initial energy distribution is related to the excess photon energy above the work function,^29,30^ and the peak currents are much higher than in conventional TEM; thus space charge effects are more important. Photoelectron guns are typically operated in a linear photoemission regime governed by a constant quantum efficiency, so the bunch charge is proportional to the total energy of the UV pulse, at least until it reaches saturation due to space charge effects (e.g., the Child-Langmuir limit^31,32^). After emission, the space charge effects depend on the electron density (and shape) in the photoemitted bunch.^16^

In the UEM geometry, such charges in a single bunch will result in a substantial energy broadening induced by space charge effects. By operating the instrument in near single-electron mode, space charge effects can be reduced. However, for many experiments, especially for systems with long recovery times that require measurement at low repetition rates, this mode of operation can result in unrealistic acquisition times. Many electrons per pulse may therefore be required for acquiring a high-resolution image or a diffraction pattern. For UEM instruments based on a modified thermal emission TEM geometry, it has been shown that the Wehnelt bias voltage greatly influences the energy dispersion.^8^

**FIG. 4.**
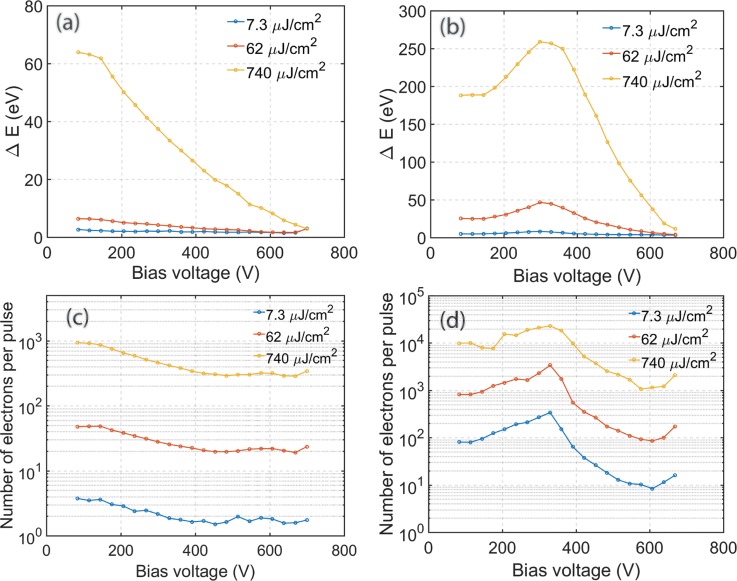
Energy dispersion of electron bunches photoemitted from **(**a**)** a guard ring cathode and **(**b**)** a truncated tip cathode at increasing UV fluence (blue 7.3 *μ*J/cm^2^, red 62 *μ*J/cm^2^, yellow 740 *μ*J/cm^2^). The corresponding average numbers of electrons per pulse measured at the retractable CCD camera are shown in panels **(**c**)** for the guard ring cathode and **(**d**)** for the truncated tip cathode.

The experimental energy dispersion from a guard ring cathode (positioned at a 700 *μ*m Wehnelt gap) and a truncated tip cathode (600 *μ*m Wehnelt gap) as a function of bias voltage at different laser fluence is shown in Figs. 4(a) and 4(b), respectively. Note that the focusing power of the Wehnelt is a function of the bias voltage, gap, as well as the cathode/Wehnelt geometry, such that the bias voltage is not a directly comparable measure of Wehnelt actions for different setups. However, since the Wehnelt focal length cannot be directly measured, we simply plot the energy spread as a function of bias voltage. For the truncated tip cathode [Fig. 4(b)], the energy spectra are collected by only considering the central photoelectrons, i.e., excluding the shank electrons by the entrance aperture to the EELS spectrometer. The energy dispersion of the electron bunches emitted from the guard ring cathode steadily decreases with increasing Wehnelt bias voltage. The truncated tip cathode exhibits more complex behavior: the energy dispersion will initially increase with Wehnelt voltage, reaching a maximum around 300 V, after which it continuously decreases. These observations are against a general intuition that a tighter focusing is expected to generate a smaller crossover and consequently more space charge effect. Furthermore, we observe a strong correlation between the energy spread and the number of electrons out of the gun, shown in Figs. 4(c) and 4(d), indicating that the space charge effect is a (more complex) consequence of electron trajectories, rather than simply the crossover size.

As mentioned above, the focusing strength of the Wehnelt increases with the bias voltage. This will influence the proportion of electrons transmitted through the limiting aperture in the anode section. For a truncated tip cathode, in the low Wehnelt bias regime, increased convergent lensing (under-focusing regime) will allow more electrons to escape through the limiting aperture at the anode. Thus, the number of transmitted photoelectrons initially increases with bias voltage. In the tip cathode geometry, the shank acts as a defocusing lens element, weakening the Wehnelt lens effect or making a diverging lens overall at low bias voltage. However, the Wehnelt field can penetrate more easily compared to large flat cathodes, and Wehnelt focusing power increases more rapidly at high bias. Above 300 V in the present geometrical configuration, the focal point of the Wehnelt is formed before the anode, and the transmission of electrons through the limiting aperture starts to decrease by the Wehnelt bias (over focusing regime), resulting in a reduction of the number of electrons per bunch with bias voltage. For the guard ring cathode, without any shank-emitted electrons and already strong Wehnelt focusing at low bias, the number of transmitted electrons continuously decreases with increasing bias voltage as shown in Fig. 4(c). In flat guard ring cathode geometry without shank, Wehnelt always acts as a focusing lens, but due to the large flat area of cathode, the Wehnelt field cannot easily penetrate to the cathode center. Its focusing power increases less drastically with bias increase. Note that the gaps employed here are fairly large, such that the Wehnelt acts as an over-focusing lens in the typical operational bias voltage range. A high bias will tightly focus the electron beam close to the cathode, and the majority of high angular components will be filtered out by the limiting aperture(s). Figure 4(d) show that more electrons are emitted from the truncated tip cathode than the guard ring cathode shown in Fig. 4(c). This is because of the combination of a larger diameter of the present truncated surface and the shank emission. This will result in higher energy dispersion due to an increased contribution from the space charge effect. However, as can be seen in Fig. 4(a), the energy dispersion at high UV laser pulse energy is reduced with increasing Wehnelt bias voltage. With 700 V on the Wehnelt electrode, the energy dispersion appears almost independent of the UV laser power, approaching that of the almost single electron per pulse regime (neglectable space charge effect regime) even though we are generating thousands of electrons at the cathode.

From the above, we can conjecture (and verify in simulations) that the energy spread due to the space charge effect will be a nonlinear function of Wehnelt focusing. A high bias on the Wehnelt will result in a strong focusing of the beam with a short Rayleigh length, and consequently less time for the electrons to interact with each other (diminishing space charge effects) while at lower bias the focusing is weak with a long Rayleigh length and interaction time, and thus the space charge effect will become cumulated and more pronounced. At much lower bias, the focusing is very weak (or even diverging beam) and the charge density never gets high enough for the electrons to repel each other, and the space charge effect diminishes.

#### Effective source distribution

**FIG. 5.**
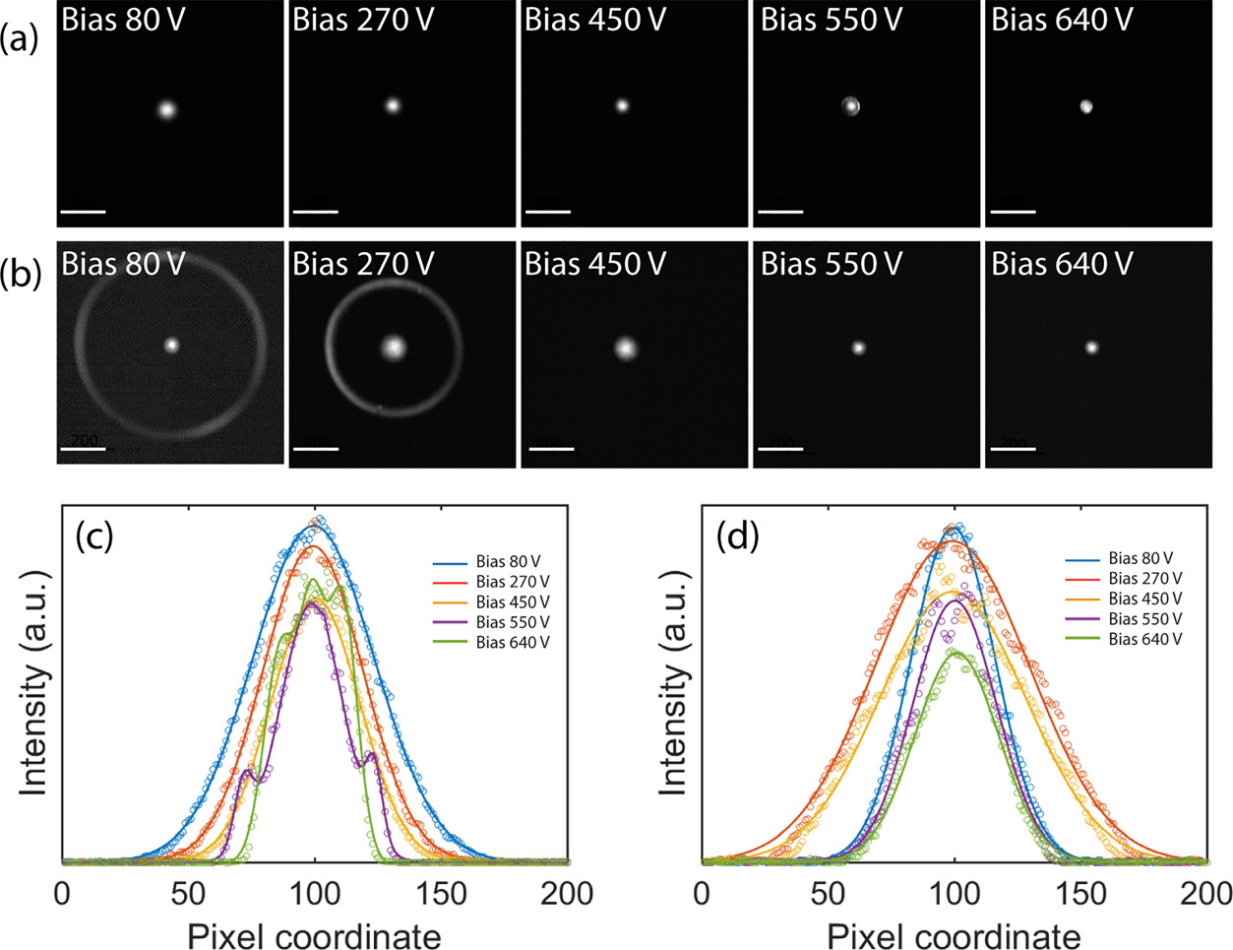
Effective source distributions of emission from (a) a guard ring cathode and **(**b**)** a truncated tip cathode at increasing bias voltage on Wehnelt. The scale bars represent 200 pixels. **(**c**)** and **(**d**)** Line profiles (200 pixels) and fitted Gaussians around the center spot showing the effective source distribution for the guard ring cathode and truncated tip cathode, respectively. The curves with shoulders in (c) are a result from a four terms Gaussian fitting. The UV laser fluence was 740 *μ*J/cm^2^.

The direct imaging (DI) method^33^ was used to analyze the effective source of the photocathodes under relevant conditions. The DI method utilizes fully spread illumination in the specimen plane so that, in the diffraction mode, the crossover in the back focal plane of the objective lens is imaged onto the camera providing a continuous spatial frequency measurement of the effective source distribution. Figure 5 shows images of the effective source distribution as measured using the DI method for the guard ring cathode and for the truncated tip cathode at different bias voltages. The UV laser fluence was 740 *μ*J/cm^2^.

For the truncated tip cathode, a ring surrounding the central emission spot can be observed at low Wehnelt bias (i.e., 80 V and 270 V). This ring is associated with shank-emitted electrons. In the corresponding images taken from the guard ring cathode [Fig. 5(a)], no such ring is present, illustrating how the cathode surface geometry and graphite mantle effectively eliminate the shank emission. Figures 5(c) (guard ring) and 5(d) (truncated tip) show fitted line profiles through the center of each source image. The beam width analysis reflects the size of the crossover at the sample plane for a given set of condenser lens settings. Qualitatively, a smaller crossover indicates a lower beam emittance, and thus better spatial coherence for a given illumination spot size as well as better potential resolution for scanning modes, provided that the reduction in emittance does not come with a dramatic reduction in current [at constant brightness, current is proportional to the product of emittance x and emittance y (Ref. 34)].

For the guard ring cathode, the transmitted intensity initially decreases with increasing Wehnelt bias voltage and the beam gets smaller. At 550 V on the Wehnelt, distinct shoulders appear on each side of the central peak, and the transmitted intensity reaches a local minimum. At 640 V, the intensity increases slightly and the shoulders move closer to the central peak. The shoulders identify the formation of a halo around the central beam, due to the high aberration of Wehnelt, similar to what has been reported in Ref. 11. The halo can also be recognized directly in the 550 V bias panel in Fig. 5(a). The intensity profiles from the truncated tip cathode show that the beam initially becomes broader with increasing bias voltage, reaching a maximum width at around 270 V [Fig. 5(d)]. However, at even higher bias the beam once again becomes narrower, while the transmitted intensity gradually decreases. In contrast to the guard ring cathode, there is no halo at intermediate bias.

### Finite element simulations

#### Spatial distribution of electrons

As evident from the experimental result presented above, both the cathode geometry and the Wehnelt bias influence the spatial and temporal resolutions as well as the number of electrons transmitted into the column. To establish which factor dominates the electron beam performance, we employed finite element simulations for the trajectories of individual photoelectrons travelling from the cathode through the accelerating section, past the limiting aperture, and into the TEM column. The 3D simulations trace individual trajectories of all electrons in the photo-emitted bunches to establish the positions of every electron allowed through the limiting aperture in the TEM column. In the model, the Wehnelt gap is set to 750 *μ*m for the guard ring cathode and 650 *μ*m for the truncated tip cathode so that the cut-off voltage (where the electric field is zero at the cathode surface as shown in supplementary material, Fig. S1) is the same as in the experiments.

**FIG. 6.**
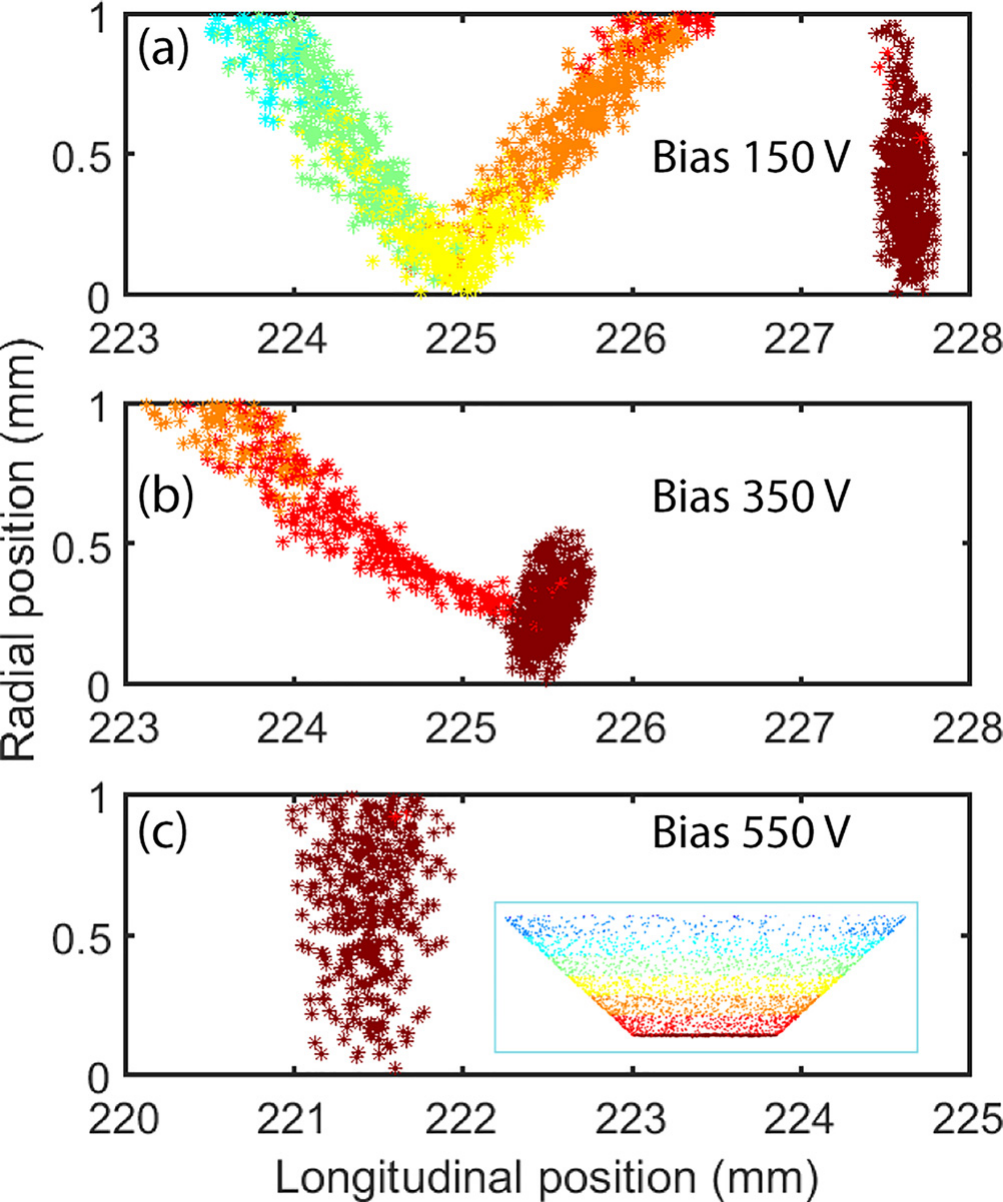
Electron positions after 1300 ps flight time for a 2000 electron bunch photoemitted from a truncated tip cathode. The simulated Wehnelt bias was set to: **(**a**)** 150 V, **(**b**)** 350 V, and **(**c) 550 V. The inset in (c) shows the initial lateral position of the electrons on the surface of a tip cathode (color-coded).

The inset of Fig. 6(c) shows, in color code, the emission position of every photoelectron at the truncated tip electrode. Red color indicates electrons from the central truncated surface of the tip, while yellow and blue indicate shank-emitted electrons exceedingly distant from the flat truncated surface. Figure 6 illustrates the longitudinal and lateral position of every photoelectron after 1300 ps of travel, at a position corresponding to immediately after the limiting aperture. The simulations show that at low Wehnelt bias, the shank-emitted electrons are temporally separated from the photoelectrons in the central beam. The temporal difference decreases with increasing Wehnelt bias voltage, but unfortunately, this comes at the expense of increases in the temporal spread of the central electron beam. At a Wehnelt bias, when the shank-emitted electrons are rejected by the limiting aperture, the temporal resolution exceeds several ps. The simulation is in good agreement with the experimental results of Bücker *et al.*^11^ and shows that truncated tip cathodes are restricted to operate at high Wehnelt bias, where shank-emitted electrons are completely suppressed. Hence, since the temporal spread of the photoelectron pulse deteriorates with increasing bias voltage, as shown in Fig. 3, truncated tip cathodes are not an optimal choice for ultrafast electron microscopy.

**FIG. 7.**
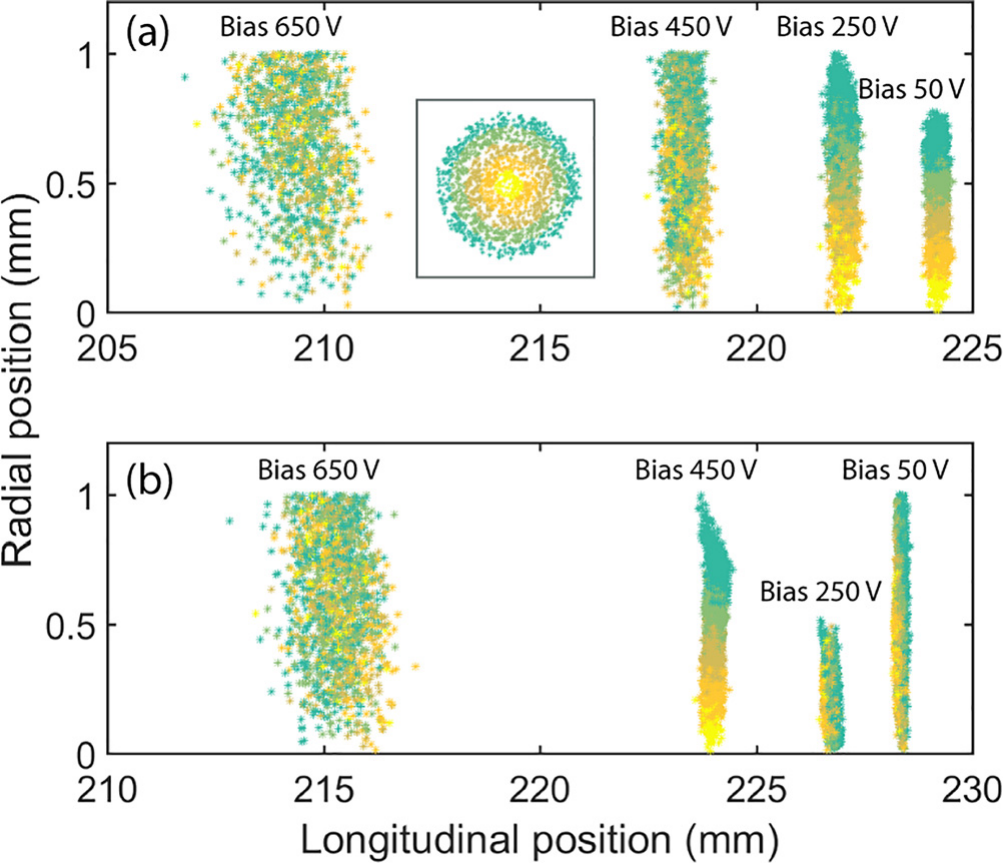
Electron positions after 1300 ps flight time for a 2000 electron bunch photo-emitted from **(**a**)** a guard ring cathode (750 *μ*m gap) and **(**b**)** a truncated tip cathode (650 *μ*m gap). For the truncated tip cathode, only electron emission from the flat central truncated area was considered (shank-emitted electrons were omitted). The figure shows the influence of the Wehnelt bias on the dispersion of the electron bunch. The inset show the initial lateral distribution of the photoelectrons on the central flat surface of the cathode (in the relevant color-code).

For the guard ring cathode geometry, the complications associated with shank emission are eliminated. Figure 7 shows the simulated longitudinal and radial positions for photoelectrons in bunches after traveling 1300 ps from a guard ring cathode [Fig. 7(a)] and a truncated tip cathode [Fig. 7(b)] under increasing Wehnelt bias voltage. In the simulation, each electron bunch contained 2000 electrons and shank-emitted electrons were ignored in the simulation of the emission from the truncated tip cathode. The inset in Fig. 7(a) shows a color-coded distribution of initial lateral photoelectron positions on the flat (truncated) surface for both cathodes. The photoelectrons are identified according to their radial position from yellow (central) to green (perimeter). The simulated electron distributions show how the lateral distribution changes and how the longitudinal spread of the electron pulse increases with the Wehnelt bias voltage, i.e., the temporal duration of the electron pulse increases with the bias voltage. For the guard ring cathode, the lateral spread increases with the increase of bias voltage, a reflection of the beam becoming more divergent at high bias voltage. At 450 V, some electrons are cut by the limiting aperture (with a radius of 1 mm). For the guard ring cathode, the focal length of the Wehnelt is shorter than the distance to the limiting aperture. An increase in bias voltage results in an even shorter focal length and a more divergent beam at the position of the limiting aperture. However, for a truncated tip cathode, the lateral spread at the limiting aperture decreases when increasing the Wehnelt bias from 50 V to 250 V. This is because the focal length of the Wehnelt for a truncated tip cathode is longer than the distance to the limiting aperture at a 50 V bias. At 250 V, the beam is focused at the limiting aperture and thus more electrons will get transmitted through the limiting aperture (for simulated throughput ratio as a function of bias, see supplementary material, Fig. S2). At any further increase to the Wehnelt, the beam will become more divergent. These simulation results are consistent with the experimental result shown in Fig. 4. The change in beam trajectory with bias voltage affects both the space charge effect and throughput. It can also be inferred that the guard ring cathode exhibits stronger focal ability than truncated tip cathodes at low bias voltage at the current Wehnelt gap. This is also shown in the simulated electron trajectories in Fig. S5, supplementary material.

#### Temporal spread of electron bunches

**FIG. 8.**
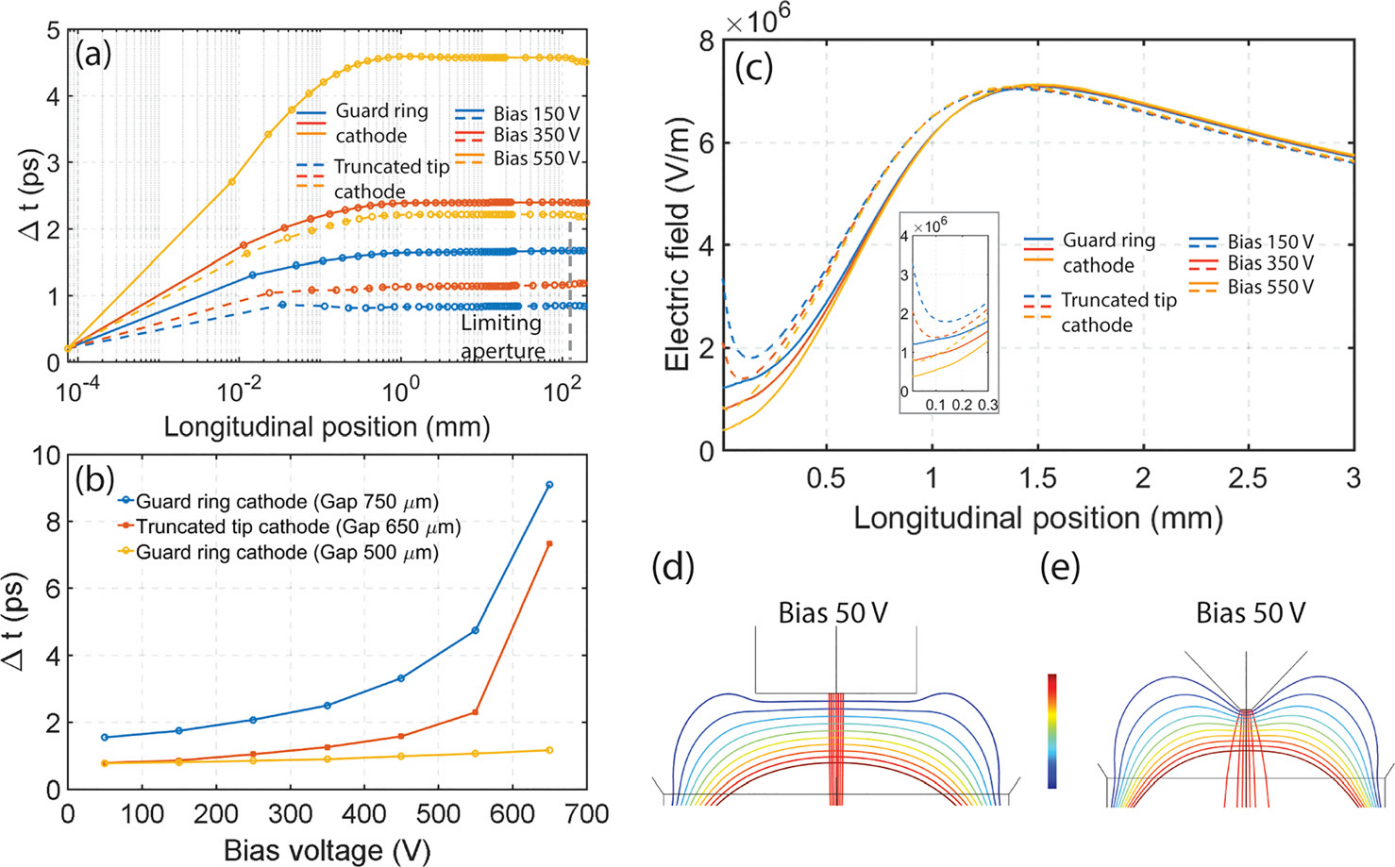
**(**a**)** Changes in temporal dispersion as a function of flight distance for electron bunches emitted from guard ring and truncated tip cathodes (only electrons emitted from the truncated surface is considered). The gray dashed line indicates the position of the limiting aperture. **(**b**)** The simulated FWHM temporal width of an electron pulse as a function of bias voltage for a guard ring cathode at 750 *μ*m and 500 *μ*m gap, and a truncated tip cathode at 650 *μ*m gap. **(**c**)** The influence of the Wehnelt bias on the extraction field in the longitudinal direction around the cathode surfaces. **(**d**)** and **(**e**)** Equipotential lines and beam trajectories around the guard ring cathode and truncated tip cathode, respectively. The range of the color scale bar is from −200.0 kV to −199.5 kV for both kinds of cathode. In all panels except (b), the guard ring cathode is placed at a 750 *μ*m gap and the truncated tip cathode at a 650 *μ*m gap.

The simulated temporal spread as a function of electron flight distance is shown in Fig. 8(a), for electron bunches photoemitted from both guard ring and truncated tip cathodes. Each simulated bunch contains 2000 electrons and the temporal spread is calculated from the longitudinal FWHM of the simulated electron bunch divided by the average velocity of the electrons. From the results, it is clear that the temporal bunch broadening takes place near the cathode surface and mainly stems from the initial energy and angular spread in the photoemission process.^35^

Electron bunches with different initial states were released from the cathode surfaces and traced during their flight path to determine the influence of initial state variables on the final temporal dispersion. The initial conditions of the simulated electron pulses are listed in Table I. The initial energy spread ΔE_i_ (standard deviation) is set to be either 0.8 eV (similar to most favorable experimental conditions) or 0 eV. The photoemitted angle distribution was selected to satisfy a Lambertian distribution (case 1, 3, and 5) or alternatively all electrons were emitted normally from the cathode surface (cases 2 and 4). In cases 1 to 4, the emitted electrons obey Gaussian spatial distribution on the cathode surface in accordance to the exciting laser power density, while in case 5, all the electrons are released from the central point of the cathode. Electron-electron interaction was switched off in the present simulation. The truncated tip cathode was positioned at a 650 *μ*m gap. The guard ring cathode was positioned both at 750 *μ*m (at a similar cut-off voltage as the tip cathode) and at 500 *μ*m (at a similar surface extraction field as for the tip cathode at a 50 V bias). The results from the simulations at Wehnelt bias at 50 and 650 V are listed in Table I. The temporal broadening is affected by the local extraction field, the initial energy spread, and the angular distribution. In the ideal case 4 with no initial energy spread and all electrons emitted normally from the cathode surface, the simulation shows the best temporal resolution for both types of cathode when the bias voltage was kept at 50 V. This implies that when choosing the cathode material, we should consider not only the work function but also the angle distribution of the photoemission. As an example, the (010) and $\left(1\bar{1}0\right)$ surfaces of LaB_6_ have different photoemission angle distributions as shown in Ref. 22. For both cathodes, we know from comparing case 1 with case 5 that different lateral photoemission positions only show limited contribution to the temporal resolution (i.e., low spherical aberration).

**TABLE I.** Simulated temporal spread of electron bunches with different initial states and instrumental conditions. The guard ring cathode was positioned both at 750 *μ*m and 500 *μ*m gaps, the truncated tip cathode at 650 *μ*m, and ΔE_i_ is the initial energy spread.

Case No.	ΔE_i_ (eV)	Angle distribution	Electron emission spatial distribution	Δt (Bias 50 V)			Δt (Bias 650 V)		
				Guard ring cathode (750 *μ*m)	Guard ring cathode (500 *μ*m)	Truncated tip cathode (650 *μ*m)	Guard ring cathode (750 *μ*m)	Guard ring cathode (500 *μ*m)	Truncated tip cathode (650 *μ*m)
1	0.8	Lambertian	Gaussian	1.4 ps	0.7 ps	0.7 ps	8.0 ps	1.2 ps	6.4 ps
2	0.8	Normal	Gaussian	1.0 ps	0.6 ps	0.6 ps	6.0 ps	0.9 ps	4.9 ps
3	0	Lambertian	Gaussian	1.1 ps	0.6 ps	0.7 ps	5.1 ps	0.9 ps	4.6 ps
4	0	Normal	Gaussian	0.3 ps	0.3 ps	0.4 ps	0.9 ps	0.3 ps	1.5 ps
5	0.8	Lambertian	Central point	1.3 ps	0.7 ps	0.6 ps	7.7 ps	1.1 ps	6.1 ps

From the results of Table I, we find that the temporal broadening at bias 50 V is in part due to the initial energy spread and the angular distribution of the photoemitted electrons. However, we can also observe that at a bias of 50 V, a truncated tip cathode shows better time resolution and then a guard ring positioned at a gap that results in a similar cut-off voltage. This is true for all relevant bias voltages as shown in Fig. 8(b). The simulations of the truncated tip cathode in Table I and Fig. 8(b) are however not realistic for UEM conditions since they ignore the experimentally present shank-emitted electrons. However, it is of interest to establish why emission from a flat surface of a truncated tip cathode geometry results in shorter electron bunches at low Wehnelt bias than emission from a guard ring cathode geometry. It is well known that a strong electric field in front of the cathode will benefit the temporal resolution.^35–37^ The Wehnelt bias will influence the accelerating electric field near the cathode surface and therefore the bias will affect the temporal spread of the electron bunch. Figure 8(c) shows the longitudinal accelerating electric field in the near cathode surface region under different bias voltages for a guard ring and truncated tip cathode geometries. With increasing Wehnelt bias voltage, it is evident that the electric field decreases. For a truncated tip cathode, the electric field near the cathode surface will be larger than for a guard ring cathode positioned for a similar cut-off voltage, in particular, very near the surface of the cathode. This is due to a geometrical field enhancement in the truncated tip geometry [Fig. 8(c)], which is somewhat similar to the field enhancement of the (extruded) tip source in a field emission gun.^15,17^ As shown in Fig. 8(a), the temporal broadening takes place near the cathode surface. It can be inferred that the enhancement of the electric field shown in the inset of Fig. 8(c) is the main reason why truncated tip cathodes show better temporal resolution than guard ring cathodes at a low bias voltage in the simulation shown in Figs. 7 and 8(b). However, an applied Wehnelt bias will quickly suppress the local field enhancement. For the guard ring cathode, with its more relaxed restrictions of the Wehnelt, it is feasible to increase the electric field at the cathode surface by reducing the Wehnelt gap to 500 *μ*m where the field will be similar to the truncated tip cathode (supplementary material, Fig. S1). The temporal spread of a bunch emitted from a guard ring cathode positioned at this Wehnelt gap will match the temporal spread of an electron bunch emitted from the flat area of a truncated tip cathode (Table I). Figure S3 in the supplementary material show a more complete set of simulation results of the bias and gap dependence of a guard ring cathode. The simulated temporal resolution for initial condition case 1 (Table I) is 0.7 ps at 50 V bias. This is similar to the experimentally determined result of 0.8 ps for a 10 electron pulse (supplementary material, Fig. S4).

Figures 8(d) and 8(e) show equipotential lines around the guard ring and truncated tip cathode at a Wehnelt bias voltage of 50 V. The equipotential lines close to the guard ring cathode surface [Fig. 8(d)] are almost flat, indicating a uniform electric field. The red lines representing the electron trajectories show that the beam is focused. As a contrast, the equipotential lines surrounding the truncated tip electrode [Fig. 8(e)] are curved around the tip. Such field geometry may lead to aberrations. The electron beam is also not focused at this bias voltage, which is consistent with the results in Fig. 7 and the discussion concerning Fig. 4. This also means that the central bright spot at bias 80 V in Fig. 5(b) is a virtual beam crossover. A higher voltage is required to focus the electron beam emitted from this cathode geometry. By comparing Figs. 8(d) and 8(e), we can conclude that the guard ring cathode geometry results in a higher effective focal strength of the Wehnelt. More simulated electron trajectories, as a function of Wehnelt bias, for both the guard ring cathode and the truncated tip cathode are shown in Fig. S5, supplementary material.

**FIG. 9.**
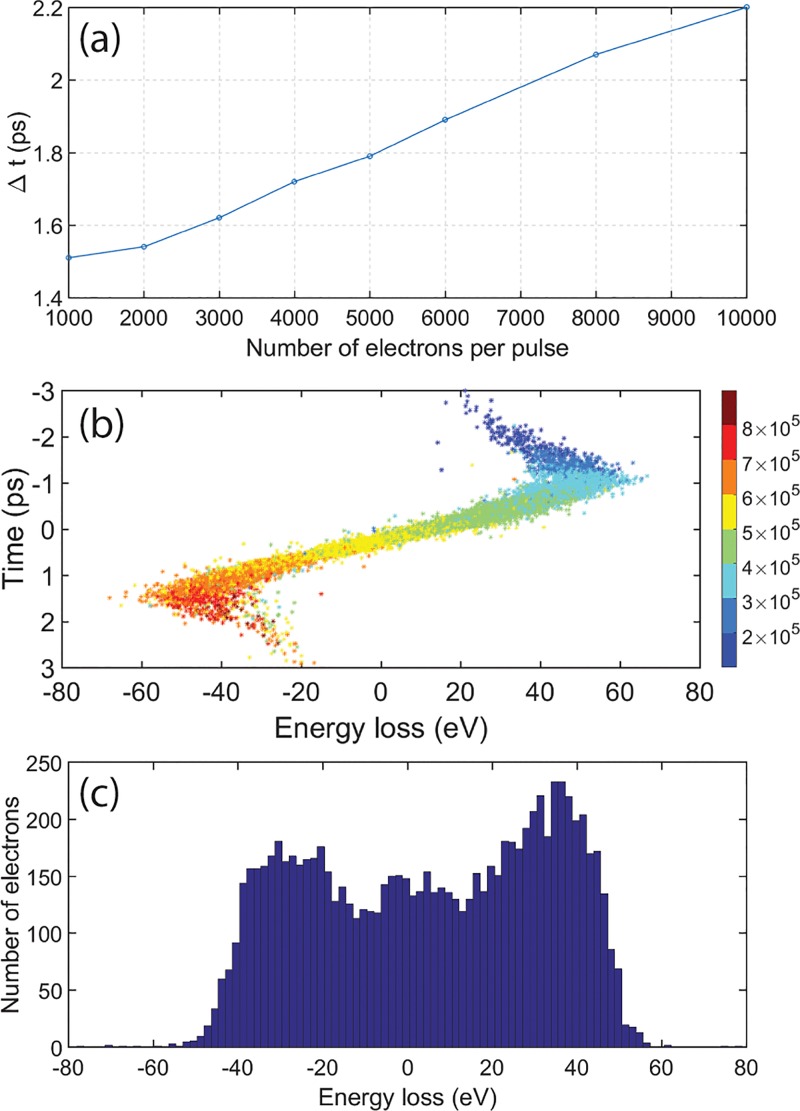
(a) Simulated temporal dispersion for electron bunches emitted from a guard ring cathode containing increasing number of photoelectrons at 50 V Wehnelt bias. (b) Simulated electron energy distribution for a 10 000 electron bunch in a relative time domain (at 50 V bias) immediately after the limiting aperture. The electrons are color coded according to their initial normal velocity in m/s. (c) Energy spectrum obtained projecting the electrons in (b) onto the energy dimension.

It is well established that the space charge effect may influence the temporal dispersion of electron bunches.^37–39^ The effect becomes increasingly important with large numbers of electrons per bunch. Figure 9(a) shows the simulated temporal dispersion of electron bunches emitted from a guard ring cathode with increasing number of electrons per bunches. The results from the simulation show that the temporal spread increases nearly linear with the increase in number of electrons per pulse within the range from 1000 to 10 000 electrons. Figure 9(b) shows the energy and time correlation of electrons in an electron pulse containing 10 000 electrons. Note that the position of the electrons within the pulse is strongly correlated with the initial normal velocity of the electron as shown by the color code in Fig. 9(b). The deviation from a linear dispersion of energy and time at both ends of the electron bunch is consistent with the results in Ref. 40. For most positions within the bunch, the charge distribution yields an electric field with a longitudinal component nearly linear to the longitudinal position, except for electrons in the front and tail parts. This aberration is a direct result of the non-monotonicity of the electric field caused by space charge and results in electrons in the very front of the bunch experiencing less acceleration than those slightly closer to the center, while electrons in the tail will experience deceleration for the same reasons. The simulated electron temporal spread is consistent with the experimental result in the PINEM experiments shown in Fig. 2(c), and the projected energy spectrum shown in Fig. 9(c) is also consistent with the overall shape of the experimental EELS spectrum, with a bimodal distribution that shows peaks at both the gain and loss energy sides.

#### Simulated normalized effective transverse emittance

From the simulated electron distribution, the normalized effective transverse emittance of the electron beam at different Wehnelt bias voltages was calculated according to Eq. (1)^41^

$$\epsilon_{n,x}=4\beta \gamma \sqrt{\bar{x^{2}}\cdot \bar{{x^{\prime }}^{2}}-{\bar{xx^{\prime }}}^{2}}.$$ (1)

**FIG. 10.**
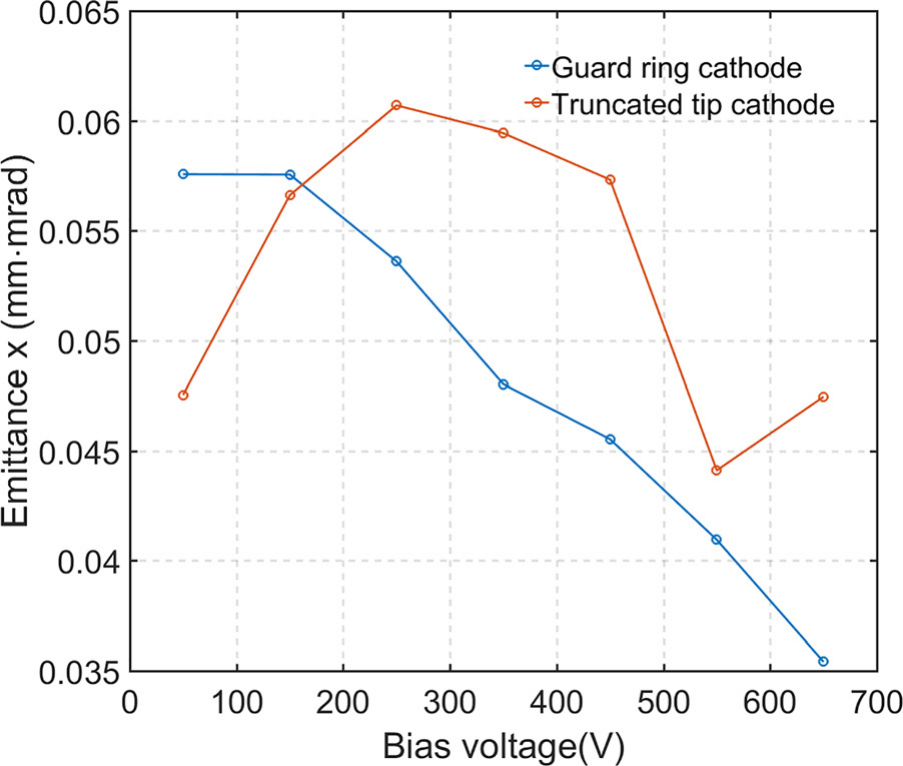
Normalized effective transverse emittance of emission from guard ring (gap 750 *μ*m) and truncated tip (Gap 650 *μ*m) cathodes under increasing Wehnelt bias voltage. Each simulated electron bunch contains 2000 electrons.

In the expression β = v/c (where v is the relevant electron velocity and c is the speed of light), γ is the Lorentz factor, x′ = p_x_/p (p_x_ is the transverse momentum). The resulting emittances are shown in Fig. 10. For a guard ring cathode, the emittance decreases with increasing bias voltage, while the emittance from a truncated tip cathode initially increases with bias and then decreases until around 600 V, which is highly correlated with the throughput (supplementary material, Fig. S2). This is due to the fact that a perfect lens does not affect emittance, but a limiting aperture does by cutting out the outer electron population at the cost of throughput.

## CONCLUSIONS

From the experimental results, it is clear that guard ring cathodes can effectively address the problem of shank-emitted electrons in the photoemission mode. This is significant since both simulations and experiments show that, at low Wehnelt bias voltage, shank emitted electrons arrive temporally shifted from electrons emitted from the central flat surfaces. As the temporal dispersion of electron bunches increases with increasing Wehnelt bias, conventional suppression of shank-emitted electrons by the Wehnelt is not a preferred solution for ultrafast analysis. The capacity of the guard ring cathode to eliminate all shank emission facilitates ultrafast imaging and diffraction experiments even at low bias voltages, allowing optimal time resolution under such experimental conditions. Compared to a disc electrode, the guard ring geometry allows for a better control of the initial beam size. The photoelectron distribution can be readily tuned by changing the size of central LaB_6_ crystal. Further, the electron beam will become less susceptible to drift in laser pointing. In the KTH UEM, using a guard ring cathode at 700 *μ*m Wehnelt gap, the temporal resolution can reach the sub-ps regime in a few electrons per bunch mode. Placing the cathode at a 500 *μ*m gap yields a resolution <800 fs and simulations show that the temporal dispersion can be further reduced by placing the cathode even closer to the Wehnelt because of the increase of the acceleration field near the cathode surface. However, for electron bunches containing several 1000 electrons, such gains may be offset by detrimental space charge effects (supplementary material, Fig. S4) due to the looser focusing conditions of the Wehnelt at smaller gaps. Loosely focused beam conditions with an extended Rayleigh length at the crossover exhibit long electron-electron interaction times and the cumulated space charge broadening can thus become significant, while for a strongly focused beam the interaction time is short resulting in a diminishing space charge effect at the crossover.

Our simulations show how the initial energy spread and the angular distribution from the photoemission process influence the temporal dispersion. A potential way to increase the temporal resolution would thus be to reduce the excess energy of the photoelectrons. This may be realized by either tuning the laser wavelength or selecting a cathode material with a work function that appropriately fits the photon energy of the laser. Addressing the broadening from the angular distribution is less trivial, but crystal truncation planes that maximize forward focusing should generally be selected.

The temporal resolution deteriorates with increasing Wehnelt bias voltage mainly because of a decrease of the electric extraction field at the cathode surface. Usually, UEM tends to be employed at low bias voltage since the temporal resolution is the most important driver for time-resolved experiments. However, another important and beneficial role of the Wehnelt electrode for the beam quality is to act as an electrostatic lens that focuses the beam. Simulations show that the geometry of the guard ring cathode enhances the Wehnelt electrode focal strength compared to the conventional cathode geometry.

However, further improvement of the UEM may require a novel gun design, including modification of the Wehnelt and cathode geometry, to realize conditions where the electron beam is tightly focused at the limiting aperture and with a high extraction field at the cathode surface.

## SUPPLEMENTARY MATERIAL

See supplementary material for simulated electric fields near the cathode surface, throughput ratio, experimental and simulated temporal resolution for a guard ring cathode placed at different Wehnelt gaps, and simulated electron trajectories as a function of Wehnelt bias.
